# Supplementary material for: Millions of Boreal Shield Lakes can be used to Probe Archaean Ocean Biogeochemistry
Source: Sci Rep. 2017 Apr 27;7:46708. doi: 10.1038/srep46708 (PMC5406836; doi:10.1038/srep46708)
Supplement: Supporting Information [file srep46708-s1.pdf]

SUPPORTING INFORMATION

Millions of Boreal Shield Lakes can be used to Probe Archaean Ocean Biogeochemistry

Schiff S. L.[1*], Tsuji J. M.[1], Wu L.[1], Venkiteswaran J. J.[1,2], Molot L. A.[3], Elgood R. J.[1], Paterson M. J.[4], and Neufeld J. D.[1*]

[1]University of Waterloo, 200 University Ave W, Waterloo, Ontario, N2L 3G1, Canada

[2]Wilfrid Laurier University, 75 University Ave W, Waterloo Ontario, N2L 3C5, Canada

[3]York University, 4700 Keele Street, Toronto, Ontario, M3J 1P3, Canada

[4]IISD-Experimental Lakes Area, 111 Lombard Ave Suite 325, Winnipeg MB R3B 0T4, Canada

*Corresponding authors: (sschiff@uwaterloo.ca, jneufeld@uwaterloo.ca)

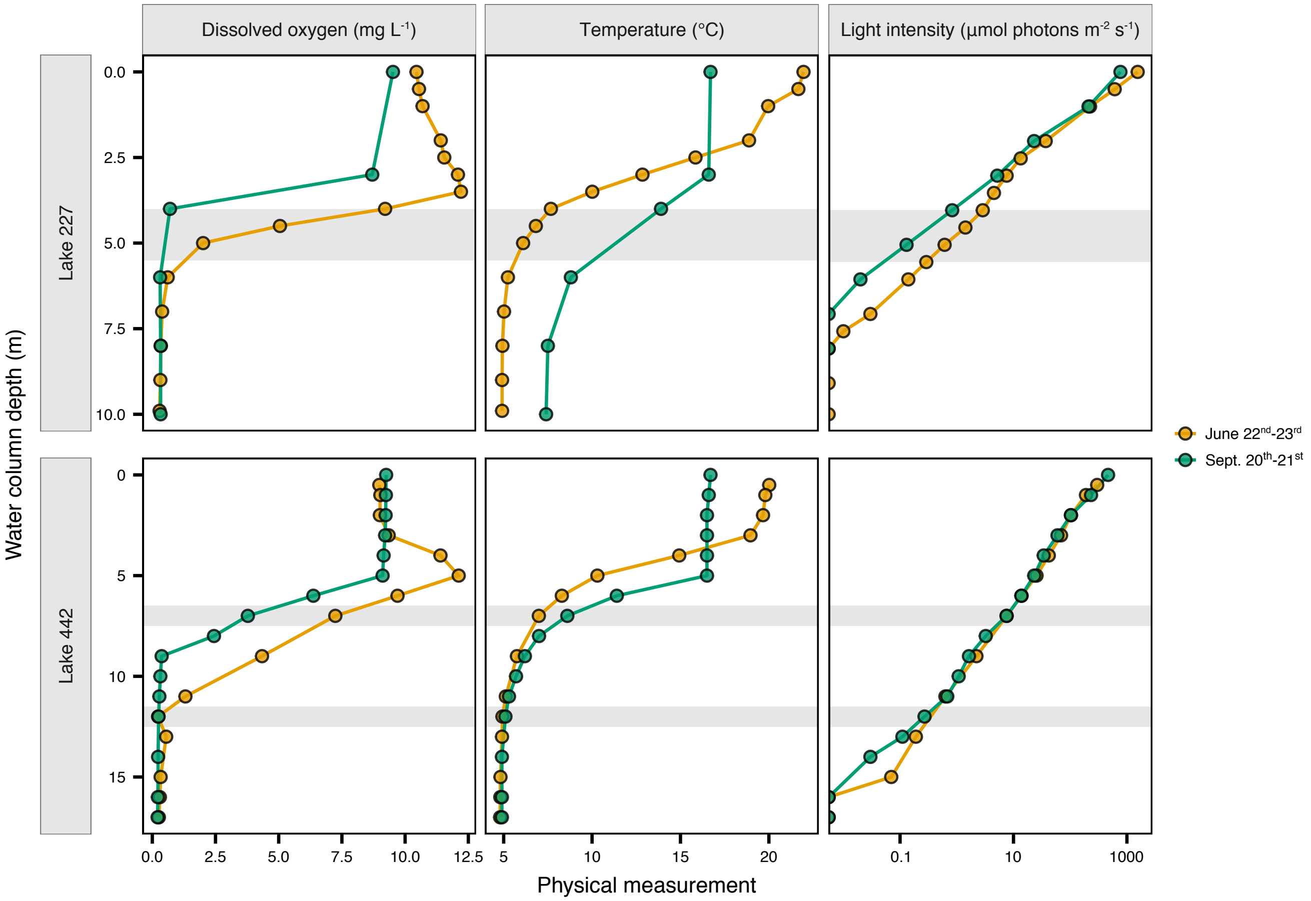

Supplementary Figure S1. Dissolved oxygen, temperature and light profiles of the L227 and L442 water columns in 2016. Grey bars represent approximate transition zones between lake layers and are identical to those shown in Figs. 1-2 (see figure captions). Exact layer boundaries at the sampling times may be estimated using the temperature and dissolved oxygen profiles shown. In both lakes, low but detectable light levels reached the top of the anoxic zone in both June and September. Light measurements below the detection limit (0.01 µmol m^{-2} s^{-1}) are plotted along the left edge of the y-axis for reference.

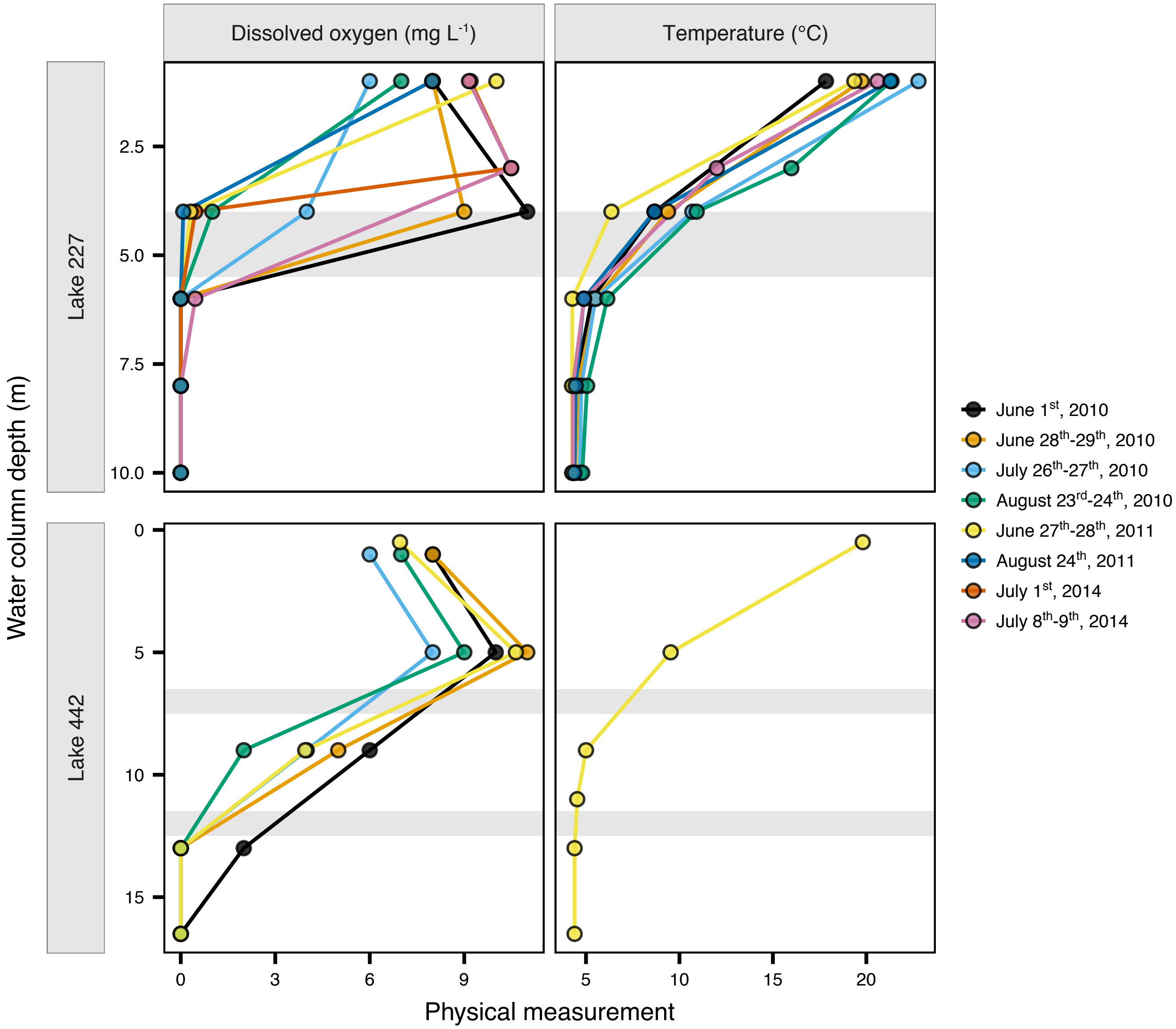

Supplementary Figure S2. Physical profiles of the L227 and L442 water columns during isotopic and molecular sampling. Grey bars represent approximate transition zones between lake layers and are identical to those shown in Figs. 1-2 (see figure captions). For comparison, actual lake zone boundaries at each time of measurement may be determined visually from temperature and dissolved oxygen data. Seasonal variations can be noted in the temperature and oxygen status of the water columns at depths near the approximate transition zones, for example for L227 at 4 m depth.

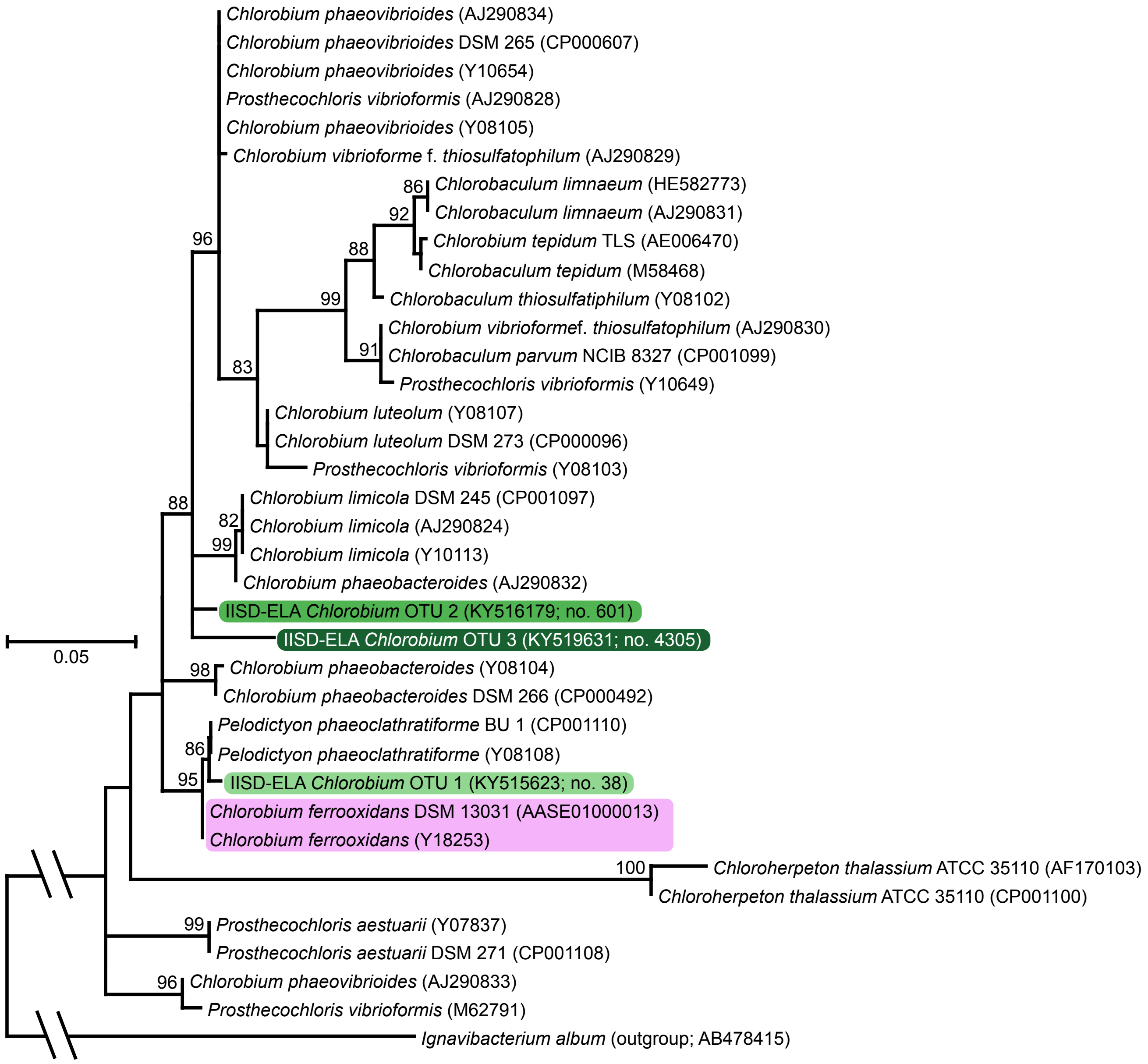

Supplementary Figure S3. Phylogenetic placement of potential photoferrotrophs within the family *Chlorobiaceae*. Reference *Chlorobiaceae* sequences represent cultured strains. Node support values, calculated using the Shimodaira-Hasegawa test, are shown where 80% or higher. Sequences highlighted in pink correspond to known photoferrotrophs, and those highlighted in green represent OTUs identified at high abundance in the water columns of L227 and L442 (Fig. 3). Importantly, IISD-ELA *Chlorobium* OTU 1 was identified at high abundance in the water columns of both lakes.

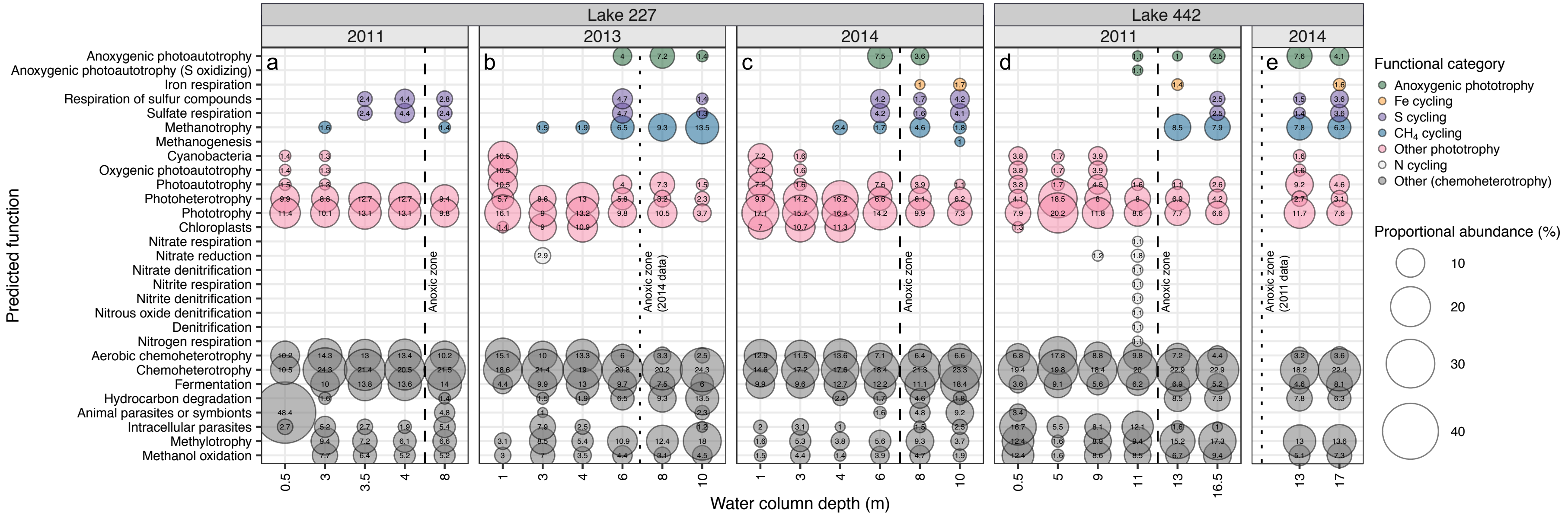

Supplementary Figure S4. Computational prediction of bacterial community function within the water columns of (a, b, c) L227 and (d, e) L442. Predicted functional roles were assigned to OTUs using FAPROTAX (*14*) for comparison to the manually curated method used for functional assignment in Fig. 3 (see Methods). For each depth sample, functional roles making up more than 1% of all predicted roles are shown as bubbles according to their proportional abundance. Importantly, the "anoxygenic phototrophy" group includes green sulfur bacteria OTUs, which may perform sulfide oxidation or photoferrotrophy (see Supplementary Fig. S3). Bacteria from the family *Comamonadaceae* (e.g. *Rhodoferax* spp., *Albidiferax* spp.) were classified as potential chemoheterotrophs or photoheterotrophs, in contrast to their classification as potential iron reducing bacteria using the manually curated approach (Fig. 3). See Supplementary Fig. S5 for a phylogeny of this family showing the placement of detected OTUs compared to known iron reducing strains. In total, 1123 of 6671 OTUs (i.e., 16.8%) could be assigned a functional role using FAPROTAX, including 48 of 74 (i.e., 64.9%) of the top ten most abundant OTUs in each sample.

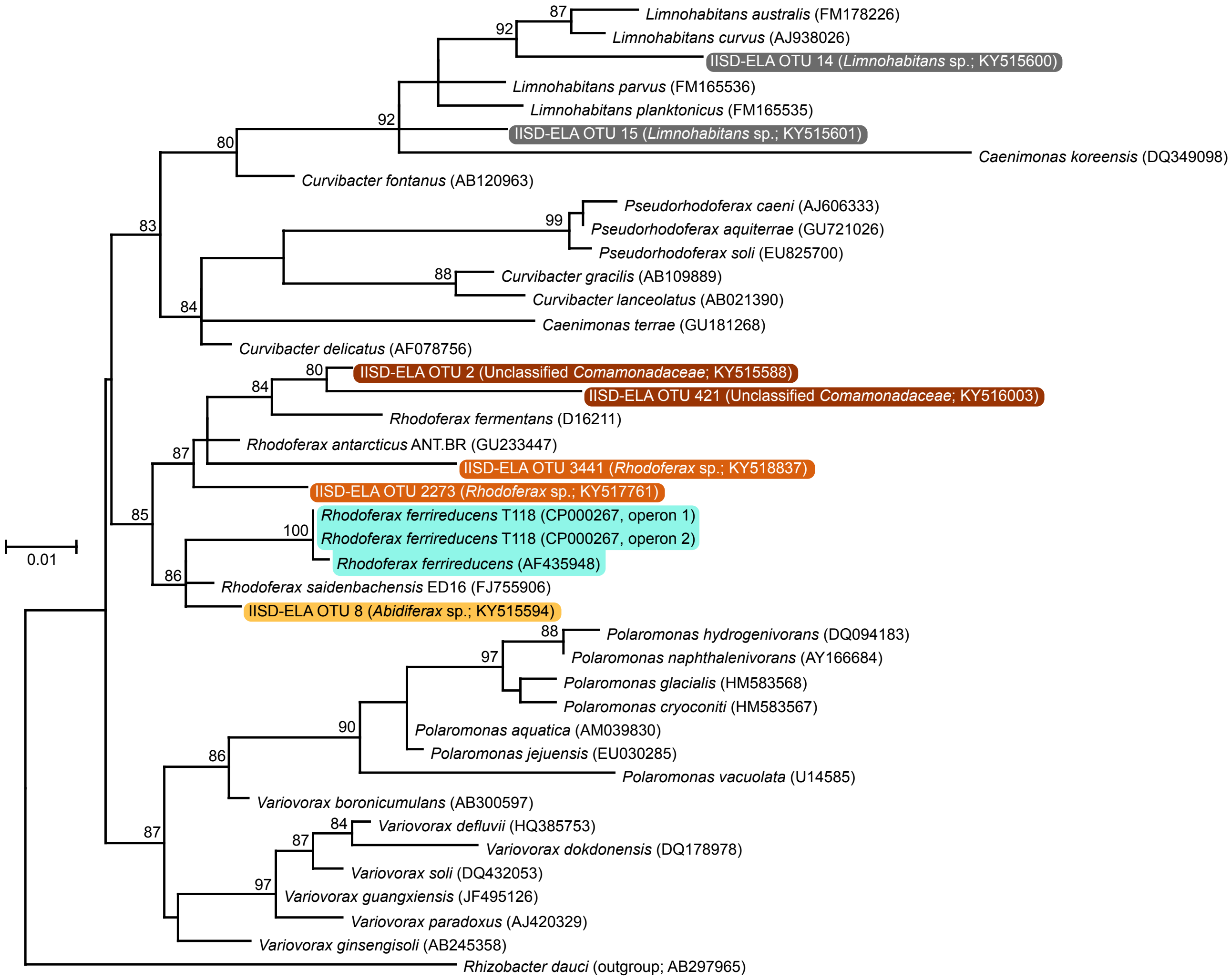

Supplementary Figure S5. Phylogenetic placement of potential iron reducing bacteria within the family *Comamonadaceae*. Reference *Comamonadaceae* sequences represent cultured strains from a monophyletic subset of the family (see Methods). Node support values, calculated using the Shimodaira-Hasegawa test, are shown where 80% or higher. Sequences highlighted in blue correspond to known iron reducing bacteria, and those highlighted in yellow or orange represent OTUs identified at high abundance in the water columns of L227 and L442 that were assigned a potential role in iron reduction using the manual annotation approach (Fig. 3; colours match those in figure). Two *Comamonadaceae* OTUs at high abundance that were manually classified as likely chemoheterotrophs are highlighted in grey for comparison.

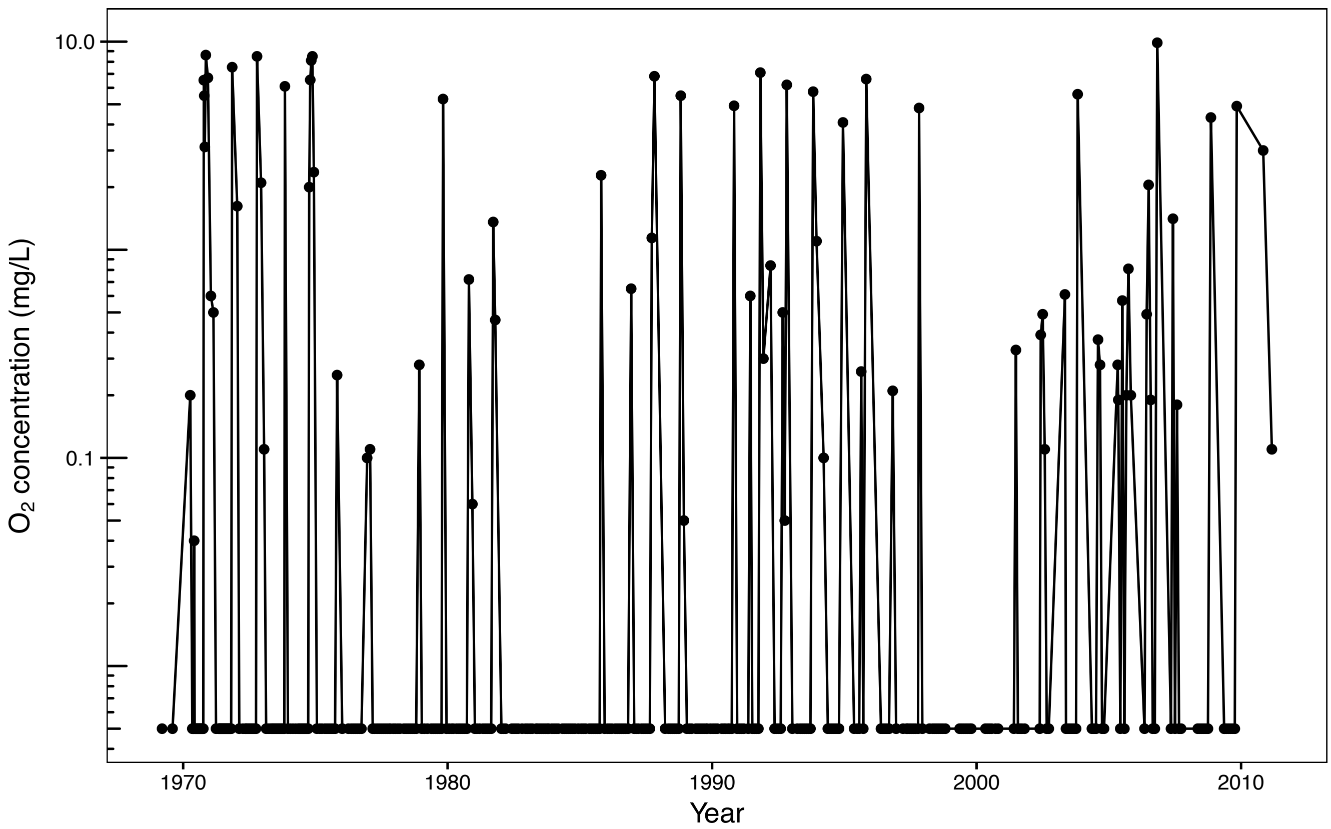

Supplementary Figure S6. Dissolved oxygen at 8 m depth in the L227 water column from 1969 to 2011. The detection limit for O_2 is 0.005 mg O_2 L^{-1}. *Chlorobium* sequences were detected at high abundance in both 2013 and 2014 at this depth (Fig. 3). Dissolved oxygen samples were collected typically at least every two weeks in summer but were collected at most twice during the winter. Following this sampling schedule, the full extent of the typical spring and fall re-oxygenation events (overturns) in L227 may not have been measured in some years. Dissolved oxygen is typically, but not always, measured after fall overturn. Spring overturn measurements can be missed following ice-off due to logistic reasons and especially in years when temperatures warm rapidly after ice-off. Thus, the oxygen record at 8 m reflects the minimum number of re-oxygenation events at this depth.

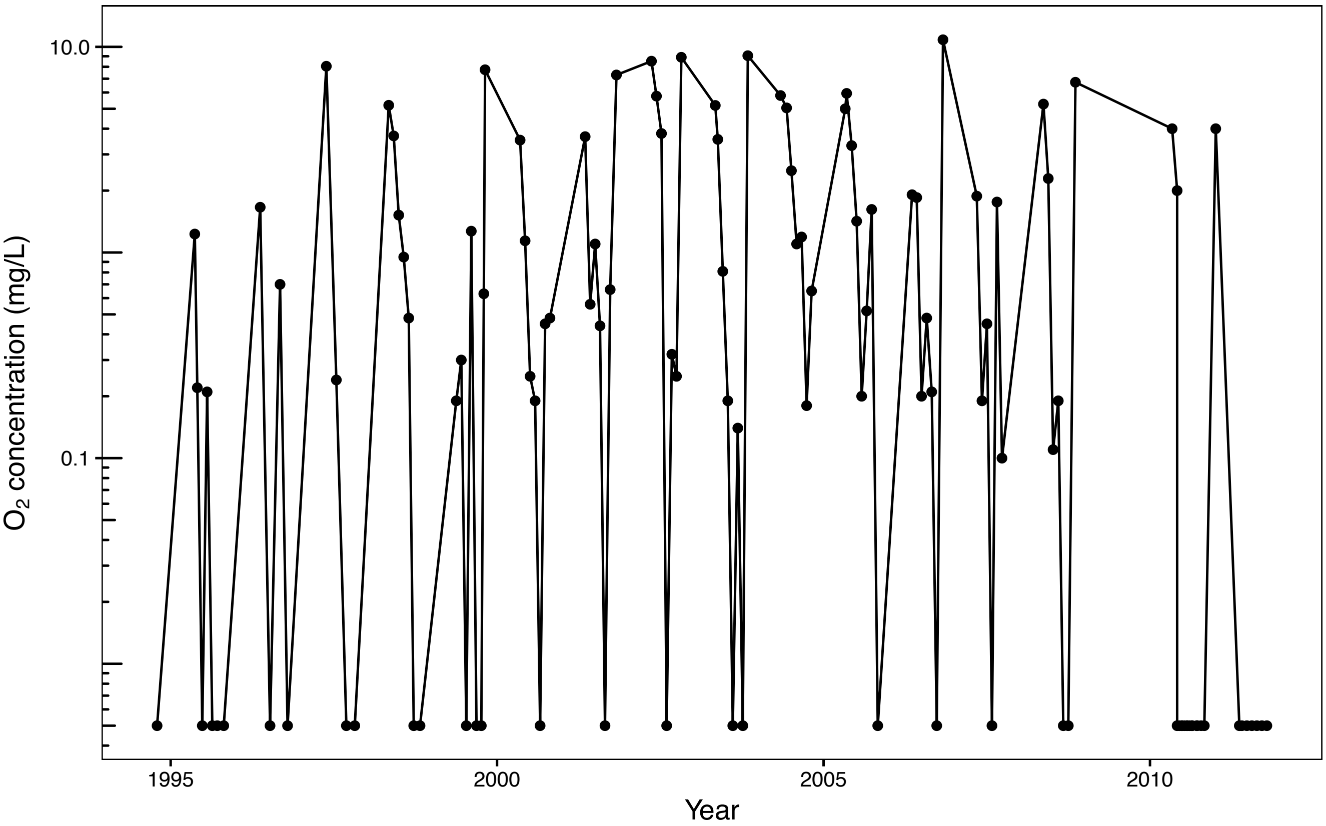

Supplementary Figure S7. Dissolved oxygen at 13 m depth in the L442 water column from 1994 to 2012. The detection limit for O_2 is 0.005 mg O_2 L^{-1}. *Chlorobium* sequences were detected at high abundance at this depth in both 2011 and 2014 (Fig. 3). Dissolved oxygen samples were collected typically once per month in summer but were collected at most once during the winter. Following this sampling schedule, the full extent of the typical spring and fall re-oxygenation events (overturns) in L442 may not have been measured in some years. Dissolved oxygen is typically, but not always, measured after fall overturn. Spring overturn measurements can be missed following ice-off due to logistic reasons and especially in years when temperatures warm rapidly after ice-off. Thus, the oxygen record at 13 m reflects the minimum number of re-oxygenation events at this depth.

Supplementary Data File S1. Rarefied hit counts of operational taxonomic units (OTU) within water column samples of L227 and L442. The OTU table (CSV format) was prepared using the software tool AXIOME2 (see Materials and Methods). Representative sequences of each OTU are included. Classification of each OTU is shown up to the taxonomic rank where the RDP classifier's confidence value fell below 50%.
